# PARADIGM-PV: a randomized, multicenter phase 4 study to assess the efficacy and safety of ropeginterferon alfa-2b in patients with low- or high-risk polycythemia vera

**DOI:** 10.1007/s00277-025-06185-5

**Published:** 2025-01-13

**Authors:** Abdulraheem Yacoub, Ghaith Abu-Zeinah, Albert Qin, Tsewang Tashi, Waleed Da’na, Weichung Joe Shih, Oleh Zagrijtschuk, Chan-Yen Tsai, Robert Geller, Norio Komatsu, Ruben Mesa, Harinder Gill

**Affiliations:** 1 Hematologic Malignancies and Cellular Therapeutics, University of Kansas Medical Center, Kansas, MO USA; 2 Division of Hematology and Medical Oncology, Weill Cornell Medicine, New York, NY 10021 USA; 3 PharmaEssentia Corporation, Taipei, Taiwan; 4 Huntsman Cancer Institute, University of Utah, Salt Lake City, UT USA; 5 King Hussein Cancer Center, Amman, Jordan; 6 Rutgers University School of Public Health, Piscataway, NJ USA; 7 PharmaEssentia USA, Burlington, MA USA; 8 PharmaEssentia Japan K.K, Akasaka Center Building 12 F, Minato-Ku, Tokyo, 107-0051 Japan; 9 Department of Hematology, Juntendo University School of Medicine, 2-1-1 Hongo, Bunkyo-Ku, Tokyo, 113-8421 Japan; 10 Department of Advanced Hematology, Juntendo University Graduate School of Medicine, 2-1-1 Hongo, Bunkyo-Ku, Tokyo, 113-8421 Japan; 11 Atrium Health, Levine Cancer Institute, Wake Forest University School of Medicine, Cancer Programs, Charlotte, NC USA; 12 Department of Medicine, School of Clinical Medicine, LKS Faculty of Medicine, the University of Hong Kong, Hong Kong, China

**Keywords:** Polycythemia vera (PV), Disease-modifying, Ropeginterferon alfa-2b, Low- and high-risk PV, Phlebotomy, Disease progression

## Abstract

Polycythemia vera (PV) is characterized by clonal hematopoietic stem or progenitor cells with constitutively active somatic mutation(s) in the *Janus kinase 2* gene. Phlebotomy (Phl) and aspirin are often used alone for low-risk PV patients. However, data from the Low-PV study demonstrated that Phl and aspirin may not be adequate for patients. Therapeutic intervention with disease-modifying treatment appears to be beneficial for patients with PV regardless of the risk category. Ropeginterferon alfa-2b (ropeg) is a novel interferon-based therapy with favorable dosing schedules. A higher starting-dose (250 µg) regimen with simpler dose titrations was found to have a potent disease-modifying effect with respect to inducing a molecular response. PARADIGM-PV is a randomized, phase 4 study with the primary goal of assessing the efficacy of ropeg at this dosing regimen in alleviating Phl-dependence in both low- and high-risk patients with PV. The secondary endpoints include complete hematologic response, molecular response, symptom improvement, maintenance of median hematocrit (Hct) values < 45% without disease progression, and safety. Patients will be randomized equally to receive either ropeg every two weeks or to continue their current treatment with Phl or other cytoreductive agents (e.g., hydroxyurea, other interferons, or ruxolitinib) as applicable. All patients will receive Phl if their Hct values are elevated to ≥45% according to the National Comprehensive Cancer Network guidelines. The study will enroll approximately 70 patients internationally, including patients in the US. This study will provide new efficacy data, measured as the ability of ropeg to reduce Phl eligibility and modify the disease.

## Introduction

Polycythemia vera (PV) is the most common classical myeloproliferative neoplasm (MPN) and is characterized by excessive erythrocyte production, panmyelosis, increased risk of thrombotic and hemorrhagic complications, and a risk of progression to myelofibrosis (MF) and acute leukemia (AML) [1, 2]. It is usually associated with a gain-of-function mutation in the gene encoding Janus kinase 2 (*JAK2*), i.e., the point mutation *JAK2*V617F of exon 14 in most cases or *JAK2* exon 12 mutations in rare cases [3–6]. However, other coexisting gene mutations or variations and epigenetic changes also exist in PV [2]. The risk stratification for thrombotic events in PV is based on age > 60 years or a history of thrombosis. High-risk patients meet either of these criteria, whereas low-risk patients do not meet either of these criteria. The cornerstone of treatment for high-risk patients includes low-dose aspirin and pharmacologic cytoreduction with or without therapeutic phlebotomy (Phl), whereas most patients with low-risk PV are treated mainly with Phl and aspirin [7]. Hematocrit (Hct) has been identified as an important risk factor for thromboembolic (TE) events. Data from the CYTO-PV study demonstrated that patients with a Hct target of ≥45% may have a higher rate of cardiovascular death and major thrombosis [8]. Therefore, Hct levels need to be managed by Phl, a commonly used clinical procedure. Studies have also shown that frequent Phl leads to iron deficiency, poor quality of life and reduced work productivity [9–11]. Although evidence of thrombotic risk reduction in high-risk patients with therapeutic cytoreduction is robust, similar evidence for low-risk PV is lacking.

### Rationale for the study

PV is usually derived from the clonal proliferation of hematopoietic stem or progenitor cells that carry an activating *JAK2* mutation with coexisting mutations, possibly together with epigenetic changes. Regardless of low- or high-risk status, abnormal stem or progenitor cells with activating mutations continue to grow and proliferate and can cause disease progression, leading to worsening symptoms, poor blood counts that increase TE risk, and transformation to MF or even AML. Elimination of neoplastic cells carrying genetic and epigenetic alterations is important for all patients with PV. Recent data from clinical studies and revised treatment guidelines also support the use of the same treatment approach, i.e., adding potentially disease-modifying cytoreduction to Phl and aspirin for both high- and low-risk PV patients.

In high-risk PV patients (aged ≥ 60 years and/or with a prior history of thrombosis), therapeutic cytoreduction in addition to Phl and aspirin is the current standard of care [12]. Hydroxyurea (HU) is often used for patients requiring cytoreductive therapy. In 2014, the JAK2 inhibitor ruxolitinib was approved as a second-line option, limited to the treatment of patients who have had an inadequate response to or are intolerant of HU [13]. Ropeginterferon alfa-2b (ropeg), a novel mono-pegylated recombinant proline-interferon (IFN) with pharmacokinetic (PK) properties allowing dosing once every 2 to 4 weeks [14–17], was then approved by the European Medicine Agency in 2019 and by the US FDA in 2021 for the treatment of both naïve and pretreated PV regardless of the risk category [18, 19]. The Phase 3 PROUD-PV with its extension CONTINUATION-PV showed that the ropeg treatment group had a superior rate of complete hematologic response (CHR) with improved disease burden compared with the HU/best available treatment group [20].

In patients with low-risk disease (age < 60 years and no prior history of thrombosis), the recommended approach is rather conservative and mainly consists of Phl and aspirin. The ELN recommends ropeg as a therapeutic option for treatment-naïve patients with low-risk PV requiring cytoreductive therapy [21]. The National Comprehensive Cancer Network (NCCN) guidelines recently listed ropeg as a preferred treatment for low-risk patients initiating cytoreductive therapy [12]. The need for therapeutic cytoreduction is recognized only for certain groups of patients [12]. Real-world data revealed that Phl does not effectively maintain the Hct of less than 45% in a large percentage of patients with PV, leaving many patients at high risk for complications from the PV and Phl procedures [22]. Over the past 20 years, the incidence of thrombosis in high-risk PV patients has decreased from 10.95% to 3.4% per patient year, whereas it has remained substantially unchanged at approximately 2.5% for low-risk PV patients, an incidence two to three times higher than that in the general population [23–25]. Furthermore, frequent Phl causes iron deficiency, and these patients experience a myriad of iron deficiency-associated side effects, including profound fatigue and exercise intolerance, which worsens quality of life and decreases work productivity. Therefore, continuing Phl procedures for a long period of time becomes challenging and a conservative approach may not be appropriate in patients with low-risk PV. Thus, alternative treatment may be needed [23, 24].

The results from the randomized Low-PV study provide a landmark framework for adding well-tolerated, cytoreductive therapy to the treatment of low-risk PV. A fixed ropeg dose of 100 µg is superior to Phl in consistently maintaining patients with low-risk PV at an Hct target of ≤ 45% in the absence of thrombotic events, progression of leukocytosis and thrombocytosis, and worsening of splenomegaly [26–28]. In 127 patients, the primary end point was met in 81% and 51% of patients in the ropeg and standard groups, respectively [27]. The responders continued the assigned treatment until Month 24 and maintained responses of 83% and 59%, respectively. This study highlighted the importance of adding a disease-modifying, cytoreductive therapy to the treatment of low-risk PV patients. Ropeg represents an IFN-alfa (IFN-α)-based therapy, and IFN-α, like its possible prototype IFN-beta (IFN-β), functions by suppressing cell cycle progression, which is accompanied by senescence entry and the loss of tumorigenesis in neoplastic cells [29]. IFN-α-based therapies have been suggested to have disease-modifying effects by increasing progression-free, event-free, and overall survival [30, 31].

Other factors are relevant to the risk of TE besides age and prior history of thrombosis and influence disease progression in PV, which further supports the addition of an agent like ropeg to the treatment of all PV patients. Elevated WBC and platelet counts, and most notably the *JAK2*V617F allele burden, have been recognized as risk factors for thrombosis and disease progression [27, 32–38]. Ropeg treatment has been shown to reduce the *JAK2*V617F burden and induce a complete hematologic response (CHR), defined as a WBC count <10 × 10^9^/L and a platelet count ≤400 × 10^9^/L, in addition to Hct <45% without phlebotomy in the previous 3 months [20].

Ropeg has been assessed at two dosing regimens that differ with respect to the starting dose and intra-patient dose titrations. The PROUD-PV study used a starting dose of 100 µg (or 50 µg for patients receiving HU) and intra-patient dose titrations with 50 µg increments once every two weeks to a maximum-recommended dose of 500 µg [20]. This dosing regimen was subsequently approved in Europe, the US and other countries or regions. An alternative regimen of ropeg, i.e., starting at 250 µg at Week 0, titrating to 350 µg at Week 2, and then 500 µg from Week 4 once every two weeks if tolerated, has also been assessed as a treatment option to accelerate hematologic and molecular responses [39]. This regimen features flexible dose adjustment according to tolerability and safety, and its schedule can be changed from biweekly to monthly when the response appears to be stabilized after one year of treatment. The regimen has been tested in several clinical studies and found to be effective and well tolerated [39–45]. Moreover, this dosing regimen is associated with an increased probability of achieving CHR and a molecular response with acceptable safety risks [46].

Existing data suggest that ropeg can provide therapeutic benefit by exerting a disease-modifying effect on disease clone(s), thereby controlling the risk factors for thrombosis and disease progression in patients with PV regardless of the risk category. The primary goal of this study is to assess the effect of ropeg in maintaining the Hct values without the Phl need, with secondary objectives including assessing the effect of ropeg treatment on CHR, mean number of Phls over two years of treatment, disease progression, occurrence of thrombotic or hemorrhagic events, symptoms, and molecular response.

### Methods

#### Study design

**Fig. 1:**
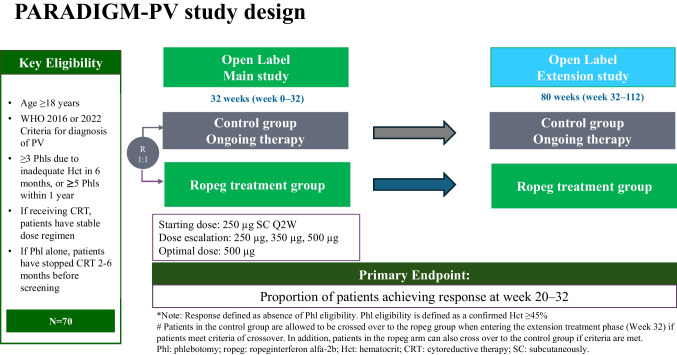
Schematic diagram of the PARADIGM-PV study design

This trial is a randomized, open-label, multicenter, two-arm phase 4 study to assess the efficacy and safety of ropeg for adult patients with PV. The study period is 112 weeks, including a main treatment phase (32 weeks), an extension treatment phase (80 weeks), and a safety follow-up phase (4 weeks) (Fig. 1). Approximately 70 patients with PV will be enrolled.

In the main phase, eligible patients will be randomized by risk classification of disease, i.e., low risk vs. high risk, at a 1:1 ratio to receive either ropeg or control treatment. In the ropeg group, patients will be treated with ropeg subcutaneously (SC) every two weeks at a starting dose of 250 µg at Week 0, followed by a dose of 350 µg at Week 2 and a dose of 500 µg starting at Week 4 if tolerated. The dose can be adjusted to the prior dose level according to tolerability and safety. The maximum recommended single dose is 500 µg every two weeks. If a dose of 250 µg leads to toxicity, dose levels of −1 (200 µg), −2 (150 µg) and −3 (100 µg) are allowed.

For the control group, patients will continue to receive the same therapy used to treat PV prior to screening, i.e., Phl and aspirin alone or Phl and aspirin plus cytoreductive agents, such as HU, other IFNs, or ruxolitinib. For both groups, Phl should be conducted when Phl eligibility is met, i.e., a confirmed Hct ≥ 45% [12].

For patients who are receiving another cytoreductive agent at screening and are randomized to the ropeg group, the agent should be discontinued with a gradual dose reduction as appropriate at or after randomization. For patients who switch from previous HU treatment to ropeg, the HU dose will be gradually reduced and its treatment will be ended in 4 weeks when the intra-patient dose titration of ropeg is completed. For ruxolitinib, the dose will be gradually reduced by 5 mg twice daily each week [47] and be discontinued in 4 weeks. The hematologic parameters and the occurrence of disease-related bleeding or major cardiovascular complications will be carefully monitored during the transition phase. If disease progression occurs, patients will be withdrawn from study. In contrast, the agent is allowed for patients randomized to the control group if it has been administered at a stable dose for at least 24 weeks before screening, and no increase in dose is planned during the study.

In the extension phase, patients in the ropeg group will continue to receive treatment every two weeks at the maximum tolerated dose level until Week 112. For the control group, patients will continue to receive ongoing therapies, e.g., Phl and aspirin only or Phl and aspirin plus prior cytoreductive therapy, until Week 112. Patients are allowed to be crossed over to the ropeg group when disease progression if they meet all the following criteria:

- Patients who are willing to receive ropeg;
- Patients who may benefit from ropeg treatment as judged by the investigator;
- Patients who experience disease progression, e.g., disease progression based on the WBC and platelet counts or other criteria that indicate the patient may benefit from ropeg treatment, as judged by the investigator.

Disease progression was defined according to Barbui et al. [26, 27] and the criteria of the International Working Group-Myeloproliferative Neoplasms Research and Treatment (IWG-MRT) [48, 49]. Specifically, one or more following characteristics are considered disease progression:

- If splenomegaly is not present by palpation at baseline, the appearance of new splenomegaly that is palpable at least 5 cm below the left costal margin according to the IWG-MRT [48];
- If splenomegaly is palpable at baseline, enlargement of a palpable spleen with an increase > 5 cm with respect to baseline according to Barbui et al. [26];
- Leukemic transformation confirmed by a bone marrow blast count of ≥ 20% according to the IWG-MRT [48];
- A peripheral blood blast content of ≥ 20% associated with an absolute blast count of ≥ 1 × 10^9^/L that persists for at least 2 weeks according to the IWG-MRT [48];
- Confirmation of post-PV MF according to the IWG-MRT [48, 49];
- A platelet count > 1000 × 10^9^/L if the baseline platelet count (i.e., the value closest to or on Day 1) was ≤ 600 × 10^9^/L or a platelet count becomes ≥ 1500 × 10^9^/L if the count at baseline was > 600 × 10^9^/L, according to Barbui et al. [26, 27].
- A WBC count > 15 × 10^9^/L if the WBC baseline count (i.e., the value closest to or on Day 1) was ≤ 10 × 10^9^/L or a WBC count ≥ 2.0 times higher than the baseline value if the count at baseline was > 10 × 10.^9^/L, according to Barbui et al. [26]
- Occurrence of clinically significant disease-related bleeding or major cardiovascular complications according to Appendix 1 of Barbui et al. [26]

For both the ropeg and control groups, a safety follow-up visit will be scheduled 28 days after the end of the extension treatment phase (Week 112 or the early termination visit). However, the study may be extended for an additional period of treatment after Week 112. If so, safety follow-up will be conducted at the end of the further extended treatment phase. Efficacy and safety will be evaluated in accordance with the protocol. Unscheduled visits will be conducted when clinically necessary as judged by the investigator. Low-dose aspirin (75–150 mg/day) is allowed to be given to patients according to the investigator’s judgement during the study. Other prophylactic antithrombotic medications may be used per the investigator’s judgment if aspirin is contraindicated.

#### Patient eligibility criteria

The key inclusion and exclusion criteria are listed in Table 1. The major inclusion criteria included age ≥ 18 years with a diagnosis of PV according to WHO 2016 or 2022 [1, 50, 51], at least three Phls within 24 weeks or at least 5 Phls within 52 weeks, WBC count ≥ 4 × 10^9^/L, and platelet count ≥ 100 × 10^9^/L. Patients who require Phl at Hct levels < 45%, have significant thrombosis (e.g., deep vein thrombosis or splenic vein thrombosis) or bleeding within 2 months, or are known to have resistance or intolerance to IFN-based therapies, are not eligible for the study.

**Table 1:** Summarizes the eligibility criteria of the study patients

Inclusion criteria	Exclusion criteria
1. Age ≥ 18 years at the time of informed consent (or other age required by local regulations) 2. PV according to the World Health Organization (WHO) 2016 or 2022 Criteria 3. At least 3 Phls within 24 weeks or at least 5 Phls within 52 weeks prior to screening due to inadequate control of Hct value [47, 48] 4. Have the following hematological values immediately prior to randomization: a. Hct <45%, b. WBC ≥4 × 10^9^/L, c. Platelet ≥100 × 10^9^/L 5. Eastern Cooperative Oncology Group (ECOG) performance status 0, 1 or 2 6. Patients receiving cytoreductive therapy must be on a stable dose for at least 24 weeks before screening with no planned dose increase [47] 7. Patients who are not receiving cytoreductive therapy must have discontinued any prior cytoreductive therapy for at least 24 weeks before screening and have recovered from any adverse events 8. Females of childbearing age, as well as all women <2 years after the onset of menopause, must agree to use an acceptable form of birth control until 60 days following the last dose of the study drug 9. Written informed consent obtained from the patient or the patient’s legal representative, and ability of patient to comply with the study requirements	1. Patients requiring Phl at Hct levels <45% 2. Clinically significant thrombosis (e.g., deep vein thrombosis or splenic vein thrombosis) or bleeding within 2 months prior to randomization 3. Post-PV MF as defined by IWG-MRT [50, 51] 4. Contraindication to PEGylated IFN or its excipients 5. Known resistance or intolerance to IFN-based therapies 6. Documented autoimmune disease (e.g., thyroid dysfunction, idiopathic thrombocytopenic purpura (ITP), scleroderma, psoriasis, or any arthritis of autoimmune origin). Patients with well-managed thyroid disease by oral hormonal replacement therapy could be enrolled 7. Pulmonary infiltrates, pneumonia, and pneumonitis at screening that, in the Investigator’s opinion, would jeopardize the patient safety or compliance with the protocol 8. Infections with systemic manifestations, e.g., bacterial, fungal, or human immunodeficiency virus (HIV), except inactive carriers of hepatitis B virus (HBV) and/or hepatitis C virus (HCV), at screening. Inactive HBV carrier is defined as the presence of HBV surface antigen and anti-HBV e antigen antibody, HBV DNA < 2000 IU/ml, and normal alanine transaminase (ALT) [52]; inactive HCV carrier is defined as the presence of HCV RNA but has normal ALT or with no clinically significant symptom as judged by investigator 9. Any investigational drug less than 6 weeks prior to the first dose of study drug or not recovered from the effect of prior administration of any investigational agent 10. History or presence of depression requiring treatment with antidepressant 11. Previous suicide attempts or at any risk of suicide at screening, in the judgment of the investigator 12. Any significant morbidity or abnormality which may interfere with the study participation 13. Pregnant or lactating females 14. History of alcohol abuse or drug abuse within the last year 15. Evidence of severe retinopathy (e.g., cytomegalovirus retinitis, macular degeneration) or clinically relevant ophthalmological disorder (due to diabetes mellitus or hypertension) 16. Significant liver (aspartate transaminase [AST] or ALT >2.5 times upper limit of normal [ULN]) or renal disease (creatinine > 2 mg/ml) 17. History of major organ transplantation 18. History or presence of clinically significant neurologic diseases, e.g., uncontrolled severe seizure disorder 19. History of malignant disease, including solid tumors and hematological malignancies (except basal cell and squamous cell carcinomas of the skin and carcinoma in situ of the cervix that have been completely excised and are considered cured) within the last 3 years

#### Study drug

The starting dose of ropeg is 250 µg at Week 0. The dose will be increased biweekly to 350 µg at Week 2 and then 500 µg beginning at Week 4 if tolerated. If tolerated, the dose will then remain fixed at 500 µg once every two weeks for the remainder of the treatment period until Week 56. The dosing schedule can be changed from biweekly to monthly if a response is achieved and stabilized as judged by the investigator. Dose adjustment, including reduction or interruption, will be performed according to tolerability and safety, as previously described [53]. Any dose adjustment must be recorded in the electronic case report form. Self-injection of ropeg is allowed after the intra-patient dose titrations of the first four weeks for patients who have demonstrated self-administration ability. The first two self-administrations will occur during normal treatment site visits after training by or under the supervision of the investigator. Patients who have successfully performed supervised self-administrations can continue self-administration in the home setting. A telephone-visit (televisit) will be performed at the scheduled date of self-administration except for the visit coincident with the onsite assessment visit. For patients who cannot self-administer the drug, a biweekly onsite visit will be scheduled instead of a televisit. For patients switching to receive ropeg treatment from the control arm, the same ropeg dosing regimen with a starting dose of 250 µg will be used.

#### Outcomes

The primary endpoint is the proportion of patients whose Hct is maintained without Phl eligibility from Week 20 until Week 32. Phl eligibility is defined as a confirmed Hct ≥45% [12]. The time of the primary endpoint measurement is the same as that previously described by others [54, 55], except the Phl eligibility follows the NCCN guidelines in this study. Patients who receive Phl due to confirmed episode of phlebotomy eligibility from Week 20 to 32 will be considered phlebotomy eligible and non-responders.

The main secondary endpoints include a comparison of the mean number of Phls from Week 0 through Weeks 32, 56, and 112, the proportion of patients with Hct values <45% from Week 0 through Weeks 32, 56, and 112, the change from baseline in the total fatigue score based on the Patient-Reported Outcomes Measurement Information System (PROMIS) short form 8a at Weeks 32, 56, and 112, the change from baseline in the total symptom score (TSS) of the Myelofibrosis Symptom Assessment Form (MFSAF) version 4.0 at Weeks 32, 56, and 112, the change from baseline in the MPN-SAF TSS at Weeks 32, 56, and 112, the occurrence of thrombotic or hemorrhagic events, the proportion of patients with CHR, and changes in the *JAK2*V617F allele burden. The occurrence of thrombotic or hemorrhagic events will be assessed throughout the entire study.

The molecular response at Weeks 32, 56, and 112, bone marrow histological remission at Weeks 56 and 112, changes in serum iron, total iron binding capacity, and ferritin at Weeks 32, 56, and 112 will also be evaluated. Safety endpoints include incidence, causality and intensity of adverse events (AEs) according to common terminology criteria for AEs (CTCAE 5.0), events leading to dose reduction or permanent treatment discontinuation, AEs of special interest (e.g., cardiovascular, thrombotic or hemorrhagic, and psychiatric events), and immunogenicity measured using an anti-ropeg antibody.

#### Sample size

The primary endpoint is the proportion of patients whose Hct is maintained without Phl eligibility from Week 20 through Week 32. Only 17% of patients were previously reported to have maintained Hct levels without Phl eligibility when receiving Phl alone or Phl plus other cytoreductive agents, i.e., HU, other IFNs, or ruxolitinib [47]. Considering potential fluctuations in the Hct value, we conservatively assumed a 20% response rate for the control group. Previously, the higher starting-dose regimen of ropeg has been evaluated in patients with PV [41], and the rate of CHR, consisting of HCT <45% without phlebotomy in the previous 3 months, a platelet count ≤400 × 10^9^/L and a WBC count <10 × 10^9^/L, was 61.2% at Week 24. Based on this result, the response rate of maintaining the Hct value without Phl eligibility is assumed to be 60% in the ropeg arm for this study. Therefore, approximately 60 patients at the 1:1 randomization ratio are needed to have 90% detection power that is significant at the 5% level, with an increase in the primary outcome measure from 20% in the control group to 60% in the ropeg group. Assuming a 10% early discontinuation rate, approximately seventy patients (35 patients per treatment group) are planned to be enrolled.

An interim analysis (IA) for sample size re-estimation (SSR) will be conducted when approximately 35 patients complete 32 weeks of treatment or withdraw from study prior to Week 32. The sample size can be adjusted upward only when the minimum of conditional power (CP) of response rate, as defined in the primary endpoint, of reaching significance at the final analysis with the original sample size is > 50%. No early stopping rule for claiming efficacy is planned. The target CP with the adjusted sample size is at least 80%, but the adjusted sample size will be capped at a maximum number that is a recommended integer that are multiples of 2, e.g. 10, 20 or 30 for minimizing the risk of revealing any CP information. When the CP with original sample size is < 50%, the study may continue without adjusting sample size. Increasing sample size when the interim result is “promising” (meaning CP at least 50% with the original sample size) is shown not to inflate the type I error rate [56–58].

#### Statistical analysis methods

The intent-to-treat (ITT) population will include only randomized patients. Analysis of the ITT population will be based on the treatment group that the patients are randomized to. The per-protocol (PP) population will consist of patients who are exposed to treatment and have all measurements needed to assess the primary endpoint without major protocol deviation. All potential protocol deviations will be reviewed prior to any interim analysis or database lock. For every potential protocol deviation, a general decision will be made whether to regard it as major or minor before locking the database. The safety population will include only randomized patients.

The proportion of patients who reach the primary endpoint will be evaluated for each arm and compared via the chi-square test. The corresponding odds ratio (ropeg/control) and its 95% confidence interval (CI) will be calculated. To determine whether the treatment effect is consistent across different risk subgroups, a subgroup analysis will be conducted to analyze the treatment effect between risk groups, i.e., low vs. high risk, with a nominal 95% CI, for the primary endpoint.

General statistical summaries will be applied to primary and secondary endpoints. For categorical endpoints, frequencies and percentages will be presented with 95% CIs. For continuous variables, the number of patients, mean, standard deviation (SD), median, lower quartile (Q1), upper quartile (Q3), minimum, maximum, and 95% CI will be presented if appropriate. For endpoints that are measured over time, the statistics will also be presented by timepoint. For time-to-event variables, the Kaplan‒Meier method will be used to estimate the cumulative distribution and associated statistics, such as the median time and CI. Missing data will not be imputed, unless otherwise specified.

AEs will be described according to the system organ classes (SOCs) and preferred terms (PTs) of the Medical Dictionary for Regulatory Activities (MedDRA) and will be graded according to CTCAE version 5.0. Descriptive statistics will be generated for the number of occurrences, number of patients and incidence of various AEs. For the immunogenicity analysis, the proportions of patients who are positive or negative for anti-ropeg antibodies and the status of neutralizing antibodies in the antibody-positive patients will be summarized.

## Discussion

The management of newly diagnosed or low-risk PV in a real-world setting often starts with Phl [59, 60]. The Low-PV study indicated that Phl alone without cytoreductive therapy was insufficient to provide adequate clinical benefits and was significantly inferior to Phl plus ropeg in maintaining low-risk patients at the Hct target of ≤45% in the absence of TE events and disease progression [27, 28]. This study highlights the clinical need for the use of tolerable, disease-modifying cytoreductive therapy in the treatment of PV, even in cases classified as “low risk”. Interestingly, only 7 of 23 (30%) nonresponding patients who switched to ropeg treatment after 12 months of therapeutic Phls met the composite efficacy endpoint at 24 months, and more frequent Phls (4.7 per patient per year) were required to maintain a Hct of ≤ 45% [27]. Moreover, modest effects were observed regarding secondary end points, likely because of the clinical and laboratory characteristics of nonresponders at baseline. Based on these observations, the subgroup of patients who switched to ropeg from Phl might need higher doses of ropeg for treatment to be effective [27, 28]. In a real-life setting, the number of patients who have received Phls for a longer period of time, such as this subgroup, may be substantially large. Patients with low-risk PV could also carry a high *JAK2*V617F allele burden [61]. Therefore, active management with ropeg at sufficient dose levels may be key for the treatment of a broad population of patients with PV.

The response outcomes observed in multiple clinical trials of ropeg confirm its clinical benefits in patients with PV. Emerging data have suggested that a higher initiating-dose regimen with accelerated dose titrations produces a CHR and molecular response earlier than the low starting-dose regimen with slow-titrations [41–44]. Although a direct comparison is not possible in the absence of head-to-head data, treatment with the higher initiating-dose regimen for 6 months led to CHR rates comparable to, or even numerically better than, those observed at 12 months of treatment in studies utilizing the low starting dose regimen with slow-titrations [41, 43]. This higher initiating-dosing regimen is also very effective in reducing the *JAK2*V617F allele burden [41, 43], which significantly impacts progression-free survival and event-free survival [31, 34]. PV is driven mostly by neoplastic cells carrying *JAK2*V617F or *JAK2* exon 12 mutations with coexisting mutations in abnormal hematopoietic lineages that lead to the overproduction of blood cells. Elimination of neoplastic cells is critical in the treatment of PV regardless of the risk category. Ropeg could directly inhibit neoplastic cells by binding to IFN receptors to induce type 1 IFN signaling, which can trigger tumor cell-intrinsic cell cycle inhibition accompanied by senescence entry and a loss of tumorigenicity [62, 63]. Higher doses of treatment lead to greater in vivo PK exposure [64], which could expose more neoplastic cells to ropeg in the hematopoietic system and induce tumor-suppressive IFN signaling at a greater level inside the neoplastic cell. Therefore, a plausible strategy for PV treatment would be to initiate ropeg therapy at sufficient dose levels, such as the higher starting-dose regimen used in this study, to rapidly achieve maximal levels of CHR and molecular response and then maintain the therapy at a less intense/stringent regimen, e.g., dosing once monthly. This approach may efficiently minimize the neoplastic cells carrying *JAK2*V617F or a *JAK2* exon 12 mutation together with coexisting mutations in the abnormal hematopoietic linages, which may maximize the suppression of disease progression and phenotypes.

In this study, patients who are randomized into the ropeg arm and have received ongoing cytoreductive therapy will discontinue the therapy with a gradual dose reduction, whereas patients randomized into the control arm will continue to receive the therapy. This design deviates from the trial that examined the effect of rusfertide on Phl eligibility [54, 55]. The discontinuation of previously ongoing cytoreductive therapy in the ropeg arm is because ropeg is a cytoreductive treatment which controls the disease and increase in spleen size effectively when administered at higher doses [43].

The novel PEGylation method provides specific PK and pharmacodynamic properties that reduce the frequency of dosing and optimize the therapeutic utility of ropeg [14–17, 65]. The reduced dosing frequency is expected to favorably contribute to patient compliance, which in turn improves therapeutic efficacy. Ropeg is a new generation of IFN-based therapy. Other products belonging to the IFN class include Pegasys®, PegIntron™, and Intron®A [66–68]. Ropeg has been officially approved for PV treatment in many countries and regions globally. Currently, most postmarketing reported adverse drug reactions (ADRs) of ropeg are consistent with those of IFN-based therapies, and no new safety information has been found to impact the current known safety profile of ropeg [69]. To minimize any safety risk, safety data will be carefully monitored throughout the study.

In summary, a strong rationale exists for adding effective and tolerable disease-modifying, cytoreductive therapy as a backbone component for all PV treatments, regardless of the risk category. The use of a higher-initiating dose regimen with flexible dose adjustments and schedule changes to implement longer intervals such as once every three to four weeks after the treatment response stabilizes has provided promising data for PV treatment with respect to efficacy and safety. This randomized, controlled phase 4 study is designed based on existing data to assess the effects of repeg on Phl eligibility as the primary endpoint. The study will also measure CHR, mean number of Phls over two years of treatment, *JAK2*V617F allele burden, blood parameters, symptoms, proportion of patients maintaining the median Hct values < 45% without disease progression, thrombotic or hemorrhagic events, and safety under the higher initiating-dose regimen.

## Data Availability

Not applicable.

## Declarations

**Ethical approval**

The study follows the principles of the Declaration of Helsinki for all human experimental investigations. The study is expected be approved by the appropriate institutional review board or ethnic committee of the participating clinical centers or hospitals. Informed consent will be obtained from all participating patients.

## Trial registration

The trial is registered at clinicaltrials.gov. The registration identifier is NCT06290765.
